# Comparison of the efficacy between Super-Path and Watson-Jones approaches in the management of early femoral head necrosis

**DOI:** 10.1097/MD.0000000000041391

**Published:** 2025-01-24

**Authors:** Yong Xu, Ping Zeng

**Affiliations:** a Guangxi University of Chinese Medicine, Nanning, Guangxi Zhuang Autonomous Region, China; b Department of Orthopedics, The First Affiliated Hospital of Guangxi University of Chinese Medicine, Nanning, Guangxi Zhuang Autonomous Region, China.

**Keywords:** COX proportional risk modeling, femoral head necrosis, Harris score, Super-Path, Watson-Jones

## Abstract

This study compares and investigates the efficacy of 2 different surgical methods for early stage femoral head necrosis and analyze the factors affecting surgical outcomes and long-term femoral head survival. A retrospective analysis was conducted on the clinical data of 48 patients (52 hips) with femoral head necrosis who underwent either the Super-Path or Watson-Jones approach from January 1, 2016, to January 1, 2024. Harris scores at multiple time points before and after surgery were compared using repeated-measures analysis of variance (ANOVA), and a COX proportional hazards model was used to analyze risk factors. The baseline data of the 2 groups were comparable (*P* > .05). There was no significant difference in preoperative Harris scores or scores at 3 and 12 months postoperatively (*t* = 0, *P* = 1; *t* = 0.719, 0.476; *P* = .716, .477). However, a significant difference was found at 36 months postoperatively (t = 2.118, *P* = .04). The preoperative stage of femoral head necrosis, patient gender, and surgical method were significant risk factors. The survival curves showed similar survival rates for the first 10 months, with no significant difference at 36 months (*P* = .5139). Both surgical approaches were effective in improving short-term hip function but did not show sustained long-term improvement. The Super-Path approach demonstrated better long-term outcomes compared to the Watson-Jones approach, influenced by surgical and temporal factors and preoperative staging. The COX model indicated that preoperative staging, female gender, and the surgical procedure were positively correlated with the risk of femoral head necrosis.

## 1. Introduction

Avascular necrosis of the femoral head mainly affects young and middle-aged patients.^[1]^ Total hip arthroplasty is considered the ultimate treatment method in the terminal stage of the disease.^[2]^ Disease progression varies among patients. Early stage options include surgery, conservative treatment, oral medication, and physical therapy^[3]^ with the aim of preserving hip joint function and delaying the loss of hip joint function as much as possible. The mainstream surgical method for early treatment involves opening the hip joint cavity, removing necrotic bone from the femoral head, grafting bone to promote new bone formation, and adding support^.[4]^ The simple method of decompression and bone grafting through trochanter drilling has been applied less clinically.^[5]^ However, there is no consensus in the medical community on the best surgical approach to enter the hip joint for bone grafting, and related studies rarely involve in-depth analysis of the relationship between the surgical approach and postoperative hip joint function.^[6]^ Therefore, our research group designed a retrospective case-control study to analyze the differences between 2 surgical approaches (the Super-Path approach and the traditional Watson-Jones approach) for bone grafting in early avascular necrosis of the femoral head. Additionally, we aimed to analyze factors related to postoperative hip joint function recovery and avascular necrosis of the femoral head.

## 2. Materials and methods

### 2.1. General data

Sample size calculation: The sample size was calculated using GPower3.1.9.7. For the statistical analysis of count data, “χ² tests” were selected, further choosing “Goodness-of-fit tests: Contingency tables.” A medium effect size of 0.5 was selected, with α = 0.05 and power = 1 – β ＝ 0.80. The degrees of freedom were (2 − 1) × (4 − 1) = 3. The calculation showed that the minimum total sample size required for this study was 44. Considering a dropout rate of 10%, the required sample size for this study was 48 cases. A retrospective analysis was conducted on 48 patients (52 hips) who were hospitalized in the Department of Orthopedics, Xianhu Hospital, First Affiliated Hospital of Guangxi University of Chinese Medicine, from January 1, 2016. to January 1, 2024. All patients signed informed consent forms for surgery, and this retrospective study was approved by the hospital’s ethics committee. All selected cases were operated on by the same chief surgeon. According to the surgical method, the patients were divided into an observation group (Super-Path approach) and a control group (Watson-Jones approach), with 24 cases in each group. There were no statistically significant differences between the 2 groups in terms of sex composition, average age, affected hip side, etiology, Ficat stage, and body mass index (BMI) (*P* > .05), making them comparable. Any differences observed in subsequent studies are more likely to reflect the effects of the intervention factors, especially the surgical methods themselves, rather than the influence of patients’ baseline characteristics. This comparability is an important basis for further analysis and interpretation of the study results (Table 1)

**Table 1:** Comparison of baseline characteristics between observation and control groups before surgery.

Variant		Super_Path	Watson-Jones	Test value	*P*-value
Sex	M	16	14	χ^2^ =0.356	*P* = .551*
	F	8	10		
age	$\bar{x}\pm s$	50.87 ± 12.37	53.41 ± 6.67	*D* = 1.155	*P* = .139*
Affected side	L	7	11	χ^2^ = 1.504	*P* = .471*
	R	15	11		
	Both	2	2		
Stage (Ficat)	I	2	3	χ^2^ = 4.778	*P* = .189*
	II	18	13		
	IIIa	1	6		
	IIIb	3	2		
Etiology	Hormone	6	9	χ^2^ = 1.129	*P* = .770*
	Alcohol	10	7		
	Trauma	5	5		
	idiopathic	3	3		
BMI	BMI = weight(kg)/height (m)^2^	23.5 (19–32.5)	25.08 ± 5.85	*w* = 24	*P* = .9423*

BMI = body mass index.

### 2.2. Surgical approaches

Observation group (Super-Path approach)^[7]:^ Patients were positioned in the lateral decubitus position, with hips flexed at approximately 45° and internally rotated by 15°. A 7 cm diagonal incision was made posteriorly and laterally from the greater trochanter. The gluteus medius was retracted anteriorly, and the gluteus maximus posteriorly. The gluteus minima was partially released to access the hip joint capsule via the space between it and the short external rotators. A U-shaped incision along the femoral neck exposed the necrotic femoral head without dislocating the hip. A 4 × 6 cm window was created at the head–neck junction to remove sclerotic necrotic bone using a bone burr and curette. Decompression was performed in the head-neck and greater trochanter areas using a 1.0 Kirschner wire. Autograft blocks from the anterosuperior iliac spine and allograft cancellous bone strips were used for the grafting.

Control group (Watson-Jones approach)^[8]^: The incision commenced at the anterior aspect of the greater trochanter, extending towards the anterosuperior iliac spine. The subcutaneous tissue and fascia were incised in alignment with a skin incision. Optimal access was achieved via blunt dissection along the anterior neck of the femur and above the gluteus minimus. Retractors were placed above and below the femoral neck on the hip joint capsule to enhance visibility. The procedure included U-shaped capsulotomy, followed by window creation in the head and neck for decompression, and combined autologous and allogeneic bone grafting.

### 2.3. Postoperative management

Post-surgery, first-generation cephalosporins were administered for 3 days to prevent infection. The sutures were removed after 2 weeks. Patients were advised to avoid weight-bearing on the affected area for 6 weeks, progressively moving to full weight-bearing over 3 months, with protected partial weight-bearing from 6 to 12 weeks.

### 2.4. Observations and statistical methods

Surgical duration, blood loss, and other relevant details were documented. Harris scores were recorded preoperatively and at 3, 12, and 36 months post-surgery. For patients undergoing total hip arthroplasty before or around 36 months, the last recorded outpatient Harris score was considered for the 36-month evaluation. Patients who did not undergo hip replacement were marked as lost to follow-up.

Data analysis was performed using SPSS version 27.0. Continuous data were analyzed using independent-sample *t*-tests, assuming normality and homogeneity of variance, or the Mann–Whitney *U* test when these assumptions failed. Categorical data were evaluated using the χ² test. Repeated measures ANOVA was used to assess Harris score changes over time, with paired *t*-tests for time-specific pairwise comparisons and Bonferroni corrections for accuracy. COX proportional hazards modeling identified factors influencing femoral head necrosis outcomes, with significance set at *P* < .05.

## 3. Results

### 3.1. Results of repeated measures ANOVA between the 2 groups before and after surgery

Through repeated measures ANOVA, we found (Table 2):

**Table 2:** Harris score repeated measures ANOVA results at multiple time points for different surgical approaches.

Group	Harris scoring system (M ± SD)			
	Pre-op Harris score (M ± SD)	Post-op 3 mo Harris score (M ± SD)	Post-op 12 mo Harris score (M ± SD)	Post-op 36-mo Harris score (M ± SD)
Super-Path	68.625 ± 11.46	71.38 ± 13.32	73.69 ± 13.57	72.7 ± 17.15
Watson-Jones	68.625 ± 13.15	68.63 ± 13.15	70.94 ± 13.01	62.04 ± 18.00
*t*	0	0.719	0.716	2.118
*P*	1**	.476**	.477**	.04*

Post-op = postoperative, Pre-op = preoperational.

* *P* < .05, indicating statistical significance.

** *P* > .05, indicates no statistical significance.

There was no difference in preoperative Harris scores between the 2 groups (t = 0, *P* = 1). At 3 and 12 months postoperatively, there were no statistically significant differences in Harris scores between the 2 groups (t = 0.719, 0.476, *P* = .716, 0.477), although the scores in the Super-Path group were higher than those in the Waterson-Jones group. At 36 months postoperatively, there was a significant statistical difference in Harris scores between the 2 groups (t = 2.118, *P* = .04).

The analysis of between-subjects factors and covariates in repeated measures ANOVA indicated: The time effect had a significant impact on Harris scores (F(3, 44) = 87.028, *P* < .001). The grouping effect did not reach statistical significance (F(1, 44) = 1.410, *P* = .241). The interaction between time and grouping reached marginal statistical significance (F(3, 44) = 2.797, *P* = .051). There was no statistically significant overall difference between preoperative and postoperative Harris scores (F = 0.227, *P* = .649). The stage had a significant impact on Harris scores (F = 3.474, *P* = .041), with significant levels as shown in Table 3.

**Table 3:** Analysis outcomes of intersubjective factors and covariant variables in repeated measures analysis of variance (ANOVA).

Covariate or factors	Time	Group	Time × group	Stage	BMI	Etiology	Harris	Age	Sex
*F*-value	87.028	1.41	2.797	3.474	0.494	0.595	0.227	1.071	0.312
*P*-value	<.001*	.241**	0.051*	.041*	.595**	0.63**	.649**	.335**	.594**

ANOVA = analysis of variance, BMI = body mass index.

* *P* < .05 indicates statistical significance.

** *P* > .05 indicates no statistical significance

### 3.2. Paired *t*-test results of Harris scores at different time points before and after surgery:

To further understand the impact of surgery on the preoperative and postoperative function of patients’ hip joints, we conducted a detailed statistical analysis of Harris scores at different time points before and after surgery. This study used paired *t*-test methods, applying Bonferroni correction to adjust the significance level. The significance level for each test was set at 0.05/6 = 0.0083 to control the Type I error rate (Table 4).

**Table 4:** Paired samples *t*-test results of Harris score between different time points before and after surgery.

Comparison group	MD	SE	95% confidence interval	*t*-value	df	*P*-value
Pre-op vs post-op 3 mo	1.375	13.15153	−5.193,2.443	0.724	47	0.472
Pre-op vs Post-op 12 mo	3.6875	13.26114	−7.538,0.163	1.927	47	0.06
Pre-op vs post-op 36 mo	1.20833	13.58027	−2.734,5.151	0.616	47	0.541
Post-op 3 mo vs post-op 12 mo	2.312	0.96	−2.591, −2.034	16.69	47	<.001*
Post-op 3 mo vs post-op 36 mo	−2.58333	16.04758	−2.076, 7.243	−1.115	47	0.27
Post-op 12 mo vs post-op 36 mo	−4.89583	16.1591	0.207, 9.587	−2.099	47	0.041*

MD = mean difference, Post-op = postoperative, Pre-op = preoperational, SE = standard error.

* *P* < .05, indicating statistical significance.

There were no statistically significant differences in Harris scores between the preoperative period and the 3-, 12-, and 36-month postoperative periods (*P* > .05).

There was a borderline statistical difference in Harris scores between the preoperative and 12 months postoperative periods (*P* = .06).

The difference in Harris scores between the 3- and 12-month postoperative periods was significant (*P* < .001).

The difference in Harris scores between the 12- and 36-month postoperative periods was also significant (*P* = .041).

The comparison between the 36- and 3-month postoperative periods did not show statistical significance (*P* = .270).

**Figure 1.**
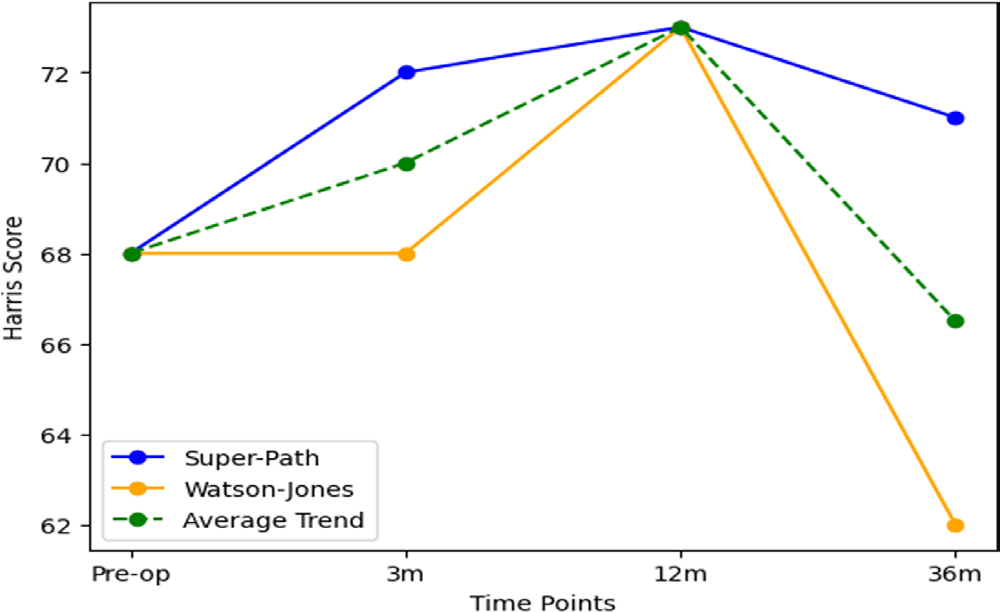
Harris score over time with significant changes highlighted.

The 2 significant differences were between 3, 12; 12, 36 months postoperatively (Table 4, Fig. 1).

### 3.3. The Cox regression model included several factors related to femoral head necrosis. The results of the model are presented (Table 5 and Fig. 2)

**Stage:** The Coef coefficient of Stage: Disease staging was 1.345 and Exp(Coef) was 3.841, indicating that for each unit increase in the surgical stage, the risk of femoral head necrosis in the patient increased by approximately 3.25 times. This indicates that the preoperative progression of disease staging is significantly associated with a worsening prognosis of femoral head necrosis.

**Table 5:** COX proportional risk model survival correlates analysis table.

Variant	Coef	Exp (Coef)	SE (Coef)	Coef-Lower 95%	Coef-Upper 95%	*Z*	*P*-value	Log2(p)
Group	−0.558	0.572	0.625	−1.78431	0.667317	−0.892	.3718	1.4271
Age	−0.001	0.998	0.029	−0.06029	0.05712	−0.052	.9578	0.0621
Sex	0.420	1.523	0.653	−0.85966	1.701629	0.644	.0519	0.9451
Stage	1.345	3.841	1.041	−0.69614	3.387997	1.291	.0196	2.3479
Op_time	0.035	1.036	0.044	−0.05234	0.123666	0.794	.4270	1.2275
Op_blood_lose	−0.019	0.981	0.050	−0.11784	0.079062	−0.386	.6994	0.5156
Etiology	0.115	1.122	0.288	−0.44963	0.68018	0.399	.6891	0.5370
BMI	0.002	1.002	0.061	−0.1182	0.122192	0.032	.9740	0.0379
Pre-Harris	0.004	1.004	0.075	−0.14426	0.152828	0.056	.9549	0.0665

BMI = body mass index, Coef = coefficient, Exp = exponential function, SE = standard error.

**Figure 2.**
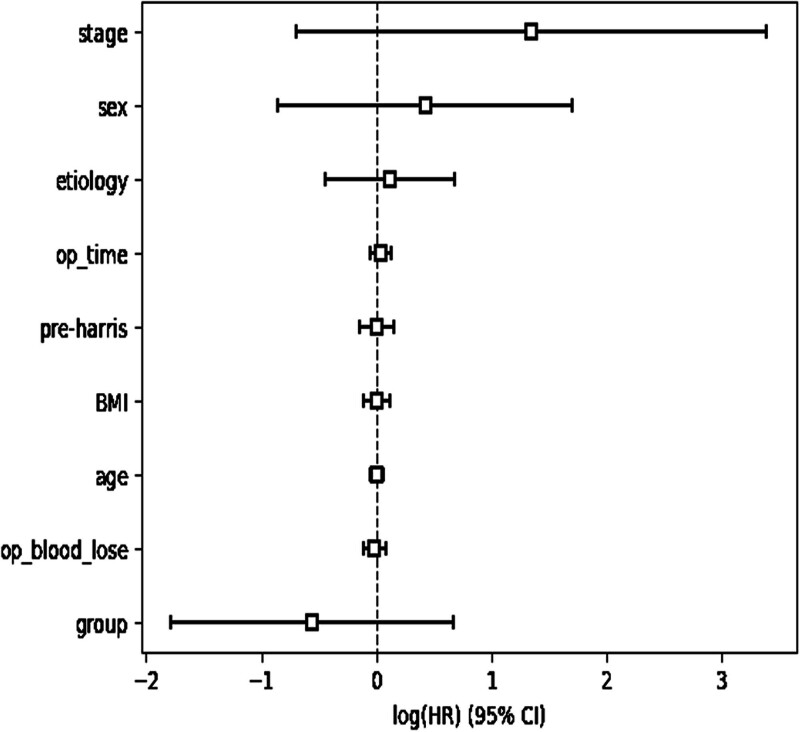
Visual representation of risk ratios of predictor variables in survival analysis.

**Group:** The coefficient was −0.558 and exp(coef) was 0.572, which means that the survival time of this group was shorter (higher risk), implying that surgery itself is also a risk factor for femoral head necrosis.

**Age**: The coefficient is close to 0, and exp(coef) is close to 1, which means that the effect of age on the outcome events of femoral head necrosis is not significant.

**Sex:** The coefficient was 0.420, exp(coef) was 1.523, and males (sex code 1 means male) had a longer survival time, which inversely suggests that females are at a higher risk of femoral head necrosis than males.

**BMI:** The BMI coefficient was close to 0, and exp(coef) was close to 1, indicating that the effect of BMI on the outcome of femoral head necrosis was not significant. Although log2(p) provides a perspective to emphasize the significance of the *P*-value, both the actual *P*-value (0.9740) and its corresponding log2(p) value indicate the same conclusion. The effect of changes in BMI on survival risk was not statistically significant. This finding suggests that BMI is not a necessary and significant factor for femoral head necrosis according to the current data and model.

Similarly, none of the remaining relevant factors were statistically significant risk factors for femoral head necrosis.

**Operative time and estimated intraoperative blood loss:** Although the operative time (*P* = .4270) and estimated intraoperative blood loss (*P* = .699) were not statistically significant, they are still noteworthy because they are usually considered to have an impact on surgical outcomes and patient recovery from a clinical perspective. This may indicate that the sample size is still insufficient or that the effects of other variables may mask their true effects.

**Figure 3.**
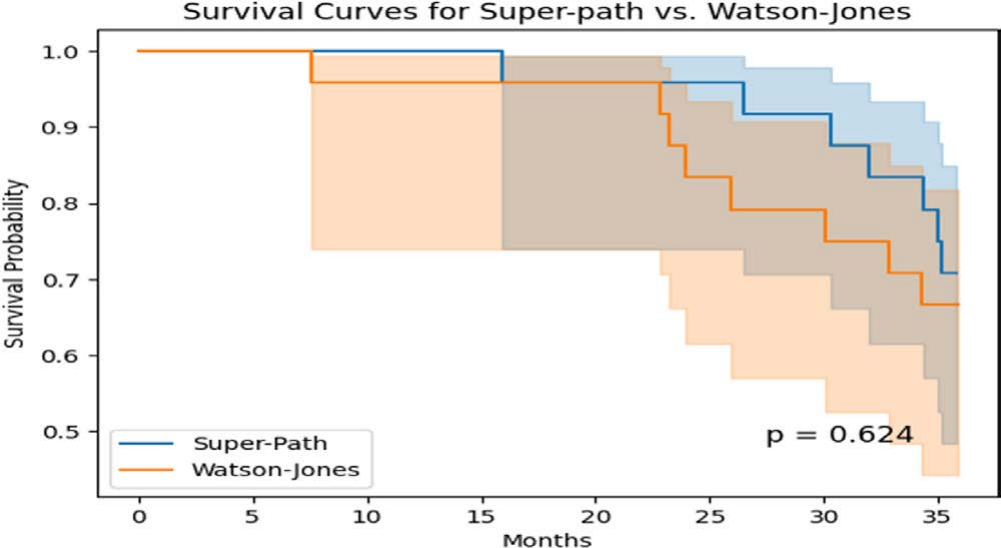
Comparison of Super-Path versus Watson-Jones surgical access on femoral survival curves.

**The overall survival curves for the 2 surgical approaches (Fig. 3**) overlapped at around 10 months postoperatively, but then there was a significant decrease in survival in the Watson-Jones group, with an even more pronounced decrease in survival in the Watson-Jones group at the time point of around 22 months, but in terms of final outcome there was no difference in survival between the 2 groups there was no statistical difference (*P* = .624).

## 4. Discussion

Drilling decompression, removal of necrotic bone, and autologous bone combined with allogeneic bone grafting are common and effective surgical methods for the treatment of early avascular necrosis of the femoral head. However, with the development of technology, clinicians have gradually abandoned the traditional surgical methods of decompression and bone grafting through the trochanteric region. Nowadays, more clinicians prefer to open the hip joint capsule under direct vision and complete bone grafting surgery with or without dislocation. This method is considered suitable for patients with avascular necrosis of the femoral head before Ficat stage IIIb.^[9]^

This study found that up to 36 months postoperatively, this bone grafting technique could maintain the patient’s hip joint function at preoperative levels, further demonstrating the clinical application value of bone grafting surgery. However, there are still multiple approaches for entering the hip joint, including the direct anterior approach, Watson-Jones approach, Smith-Peterson approach, Super-Path approach, and Kocher incision. Each approach has its unique advantages and disadvantages, but there is no consensus on which approach is superior.^[10]^ The Super-Path approach (SP) is a relatively new hip surgical approach characterized by the preservation of the muscles, ligaments, and hip capsule around the hip joint, minimizing surgical trauma.^[11]^ This method accesses the hip joint through a smaller incision without detaching or damaging the surrounding soft tissues, thereby reducing the risk of surgical complications and allowing faster postoperative recovery. In contrast, the Watson-Jones approach (WJ), although allowing direct visualization of the anterior structures of the hip joint, may result in more soft tissue damage and postoperative pain, with a longer recovery time.^[12,13]^

Through repeated measures ANOVA on Harris scores at multiple time points, this study found the following:

1. Superior long-term effects: The Harris scores of the Super-Path group were significantly higher than those of the Watson-Jones group at 36 months postoperatively, suggesting that the Super-Path method may have superior long-term effects compared to the Watson-Jones method.
2. Significant impact of time factors: Time factors had a significant impact on Harris scores, whereas the surgical method and its interaction with time did not have a significant impact, although the interaction was close to the significance level.
3. Influence of covariates: Among the covariates, only Ficat stage had a significant impact on Harris scores, while the other covariates did not.

These results indicate that different surgical methods have similar short-term effects; however, in long-term follow-up, the Super-path method may have better effects. Additionally, the Ficat stage of the patient may be an important factor affecting the postoperative Harris scores.

According to Figure 1 and Table 4, the trends in Harris scores for the Super-Path and Watson-Jones groups at each time point are as follows:

1. Short-term postoperative effects: At 3 and 12 months postoperatively, the Harris scores of the Super-Path group gradually increased, whereas those of the Watson-Jones group remained stable at 3 months postoperatively and then increased.
2. Long-term postoperative effects: At 36 months postoperatively, the Harris scores of the Super-Path group slightly decreased but remained higher than the initial values and the scores at 3 months postoperatively; the scores of the Watson-Jones group significantly decreased.

Paired sample *t*-test results showed no significant differences in Harris scores between the preoperative period and 3, 12, and 36 months postoperatively, but significant differences between 3 and 12 months, and between 12 and 36 months postoperatively.

Furthermore, the Cox proportional hazards model analysis showed that the preoperative Ficat stage of avascular necrosis of the femoral head, the surgery itself, and female sex are risk factors for the progression of avascular necrosis of the femoral head (Table 5). Although survival curve analysis indicated that the survival rates of the Super-Path and Watson-Jones methods were close within 10 months postoperatively, the difference was not significant at 36 months postoperatively (Fig. 3), suggesting no significant difference in the impact of the 2 surgical methods on the final outcome of avascular necrosis of the femoral head.

## 5. Conclusions

For early stage bone grafting surgery for avascular necrosis of the femoral head using the Super-Path and Watson-Jones approaches, although both methods can effectively improve hip joint function from 3 to 12 months postoperatively, they can only maintain preoperative hip joint function in the long-term and have no significant impact on the final outcome of avascular necrosis of the femoral head. Although the Super-Path approach shows some long-term treatment advantages, these advantages may be due to the combined effects of time, interaction of surgical methods with time, and preoperative Ficat stage of the disease. Factors affecting the final outcome of avascular necrosis of the femoral head include female sex, preoperative Ficat stage, and the surgical method itself. These 2 surgical methods themselves do not change the final outcome of avascular necrosis of the femoral head, but are related risk factors. Future research should further explore the factors affecting long-term improvement in hip joint function and the final outcome of avascular necrosis of the femoral head to guide the choice of clinical treatment plans. Choosing the Super-Path approach at an appropriate time and considering other factors related to the outcome of avascular necrosis may have long-term therapeutic effects.

## Acknowledgments

The author(s) would like to acknowledge that this research was independently conducted without any external funding or institutional support.

## Author contributions

**Data curation:** Yong Xu.

**Formal analysis:** Yong Xu.

**Software:** Yong Xu.

**Supervision:** Ping Zeng

**Writing – original draft:** Yong Xu.
